# Understanding stakeholder relationships and local context to build a community-based one health surveillance system in Guinea

**DOI:** 10.1016/j.onehlt.2025.101117

**Published:** 2025-06-21

**Authors:** Maxime Tesch, Abdoulaye Touré, Saa André Tolno, Hélène De Nys, Mathieu Bourgarel, Mamadou Alimou Barry, Mohamed Idriss Doumbouya, Marisa Peyre, Marie-Marie Olive

**Affiliations:** a CIRAD, UMR ASTRE, Montpellier, France; b ASTRE, Univ Montpellier, CIRAD, INRAE, Montpellier, France; c Institut supérieur des sciences et de médecine vétérinaire de Dalaba, Dalaba, Guinea; d Guinean Centre for Training and Research in Infectious Diseases (CERFIG), Conakry, Guinea; e CIRAD, UMR ASTRE, Harare, Zimbabwe; f Universidade Eduardo Mondlane – Campus Universitario Principal, Maputo, Mozambique; g Guinean Office of National Parks and Wildlife Reserves (OGPNRF), Conakry, Guinea; h National Directorate of Veterinary Services (DNSV), Conakry, Guinea

**Keywords:** Zoonoses, Surveillance, One health, Guinea, Participatory research, Community-based

## Abstract

In 2014, the Ebola virus epidemic that began in Guinea spread to several countries in sub-Saharan Africa, causing the deaths of almost 11,000 people. This crisis, caused in part by increased human contact with wildlife, was exacerbated by the lack of preparedness within the health sector of the affected countries, including inadequate surveillance of disease emergence from wildlife. Given that local communities face the greatest exposure to these issues, this study sought to acquire the contextual knowledge needed to set up community-based zoonotic disease surveillance. Field investigations based on a One Health approach were carried out at two sites in the Guéckédou prefecture in Guinea's forest region. Semi-structured individual interviews and focus group discussions were held with 87 members of the community. These interviews provided new information about zoonotic disease surveillance in Guinea, which we then used to map the health actors and their relationships within the community. We gathered details about the barriers they face and their concerns, such as the fundamental importance of training, a lack of legitimacy, and the differences in means allocated among the human, animal, environmental, health sectors. Some relatively unidentified stakeholders also emerged as possible communication channels. This study shows the importance of talking to the primary users of surveillance to ensure the acceptability and relevance of the surveillance system to the local community. Community members can clearly articulate their priority needs in a given context to ensure potential solutions align with those needs. The epidemiological context in Forest Guinea over the last 10 years makes this region an ideal laboratory for understanding how to tackle emerging infectious diseases in close cooperation with the people most affected.

## Graphical abstract

**Figure:**
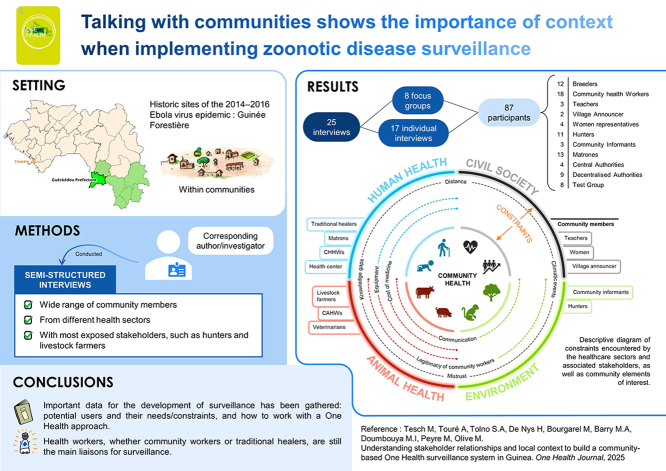

## 1 Introduction

The 2014 Ebola epidemic hit West Africa hard [1]. The first cases were reported in the prefecture of Guéckédou, located in Guinea's forest region [2]. Given the region's proximity Sierra Leone and Liberia and the daily movement of people over these borders, the epidemic also spread quickly into and across these two countries. The Guinean health system was quickly overwhelmed [3]. By the time the end of the epidemic was declared three years later, a total of 11,000 people had died [4]. The epidemic response was challenging due not only to the lack of preparedness required to manage such an epidemic in these countries [5], but also to the limited investments that had been made in risk prevention and surveillance [6].

Guinea has been resilient and learned lessons from the 2014 epidemic [7]. A national One Health platform has been in place in Guinea since 2017. This platform, which draws on a multisectoral and multidisciplinary approach, was set up to coordinate all health interventions to prevent, detect, and respond to diseases with the goal of decentralizing this platform from the national to the local levels [8]. The resurgence of Ebola in 2021 offers a concrete example of successful multisectoral implementation, from the investigation of the index case, to contact follow-up and patient management, with case numbers being drastically reduced to 16, among which 12 died [9]. But the geographical area of West Africa where Guinea is located continues to deal with numerous animal, human, and zoonotic epidemics [10]. As a result, setting up an integrated, multisectoral surveillance and investigation system to detect outbreaks and respond rapidly is a priority [11].

Communities are on the frontline when epidemics emerge: they suffer the consequences but can also play a key role in early detection [12]. It is therefore crucial to involve them in the early detection of disease emergence by including them in the surveillance system at local level through event- and community-based surveillance [6,13,14]. To be successful, community-based surveillance must be underpinned by community acceptability, strong collaboration, good communication, local ownership, and trust, and all stakeholders need to understand their roles and responsibilities [15]. Participatory methods are especially relevant in this context as they enable stakeholders to identify and propose solutions tailored to their specific constraints [[16], [17], [18]]. Previous studies have been conducted in Forest Guinea to assess the feasibility of setting up community-based surveillance. Guenin et al. (2022) noted that doctors' knowledge of diseases and recognition of clinical signs varied widely. Adapted case definitions (*e.g.* “Dehydration, diarrhoea, vomiting, or similar signs unusual in the village or in the same family”) and functional communication channels (*e.g.* discussion forum or standardized data transmission) were shown to be essential for effective surveillance [19].

This study aimed to lay the foundations of a community-based surveillance system in Forest Guinea by (i) identifying the relationships between community health stakeholders at the human–animal interface, (ii) understanding the barriers they faced in sharing information for zoonotic disease detection and (iii) drawing up recommendations and key requirements for setting up an acceptable surveillance system.

## 2 Materials and methods

### 2.1 Study location and design

**Fig. 1:**
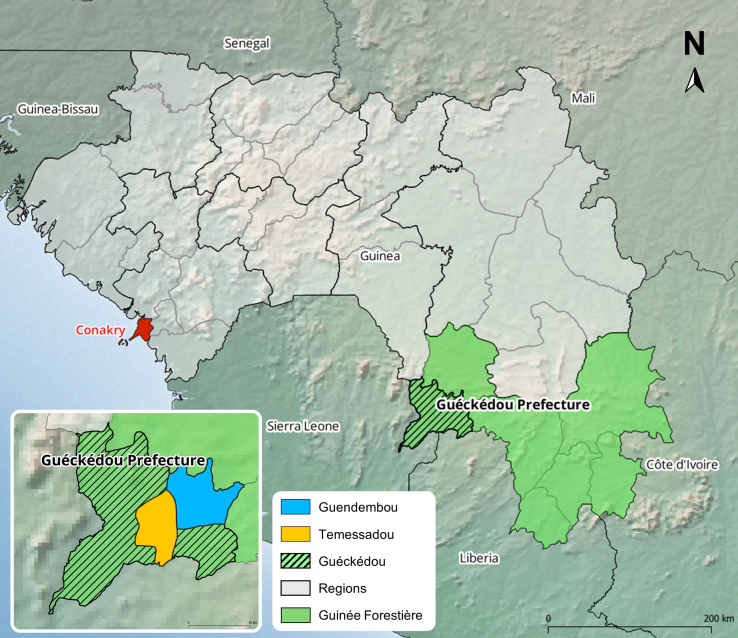
Map of the Republic of Guinea and locations of the two study sites: Temessadou and Guendembou. In red: Conakry, capital city. Map created using free and open source QGIS software (https://qgis.org), ArcGIS Hub (https://hub.arcgis.com/), Natural Earth (https://www.naturalearthdata.com/), Amerigeo (https://data.amerigeoss.org/).

We identified two study sites, the Temessadou and Guendembou subprefectures (Fig. 1), which are included in a long-term initiative to design and test a community-based surveillance system for hemorrhagic fevers of zoonotic origin in Forest Guinea as part of the EBO-SURSY project [[19], [20], [21], [22], [23]]. These sites were selected according to the following criteria: (i) proximity to the 2014 emergence location of the Ebola virus [2] (ii) accessibility from the Guéckédou prefecture, and (iii) presence of local partners trained in participatory approaches [19]. Based on previous studies and contextual data, participants were selected based on their links to the community health sector [20]. For this study, we define a community by the customs, practices, and knowledge adopted by a group of people who share ethnic characteristics and have a shared historical background [24,25]. Several key informants had been working with the research team to mobilize community members [19]. Snowball sampling and purposive sampling were used to identify new relevant participants [26,27]. The research team conducted semi-structured interviews (SSIs), which took the form of focus group discussions (FGDs) and semi-structured individual interviews (SSIIs) in April 2022 and March–April 2023 [28]. To refine the interview guidelines and facilitation techniques, a test FGD was first carried out in the Forécariah prefecture. The aim of each interview was to understand the participants' roles as community members, and their actions or considerations regarding health. The following topics were addressed during each SSII and FGD: (i) day-to-day activities and role within the community, (ii) health issues and diseases faced, (iii) information sharing, and (iv) barriers and needs. When needed, French to Kissi translation was provided by a key informant. Flow diagrams and proportional piling were used to stimulate the discussions [29,30]. Flow diagrams helped to visualize the relationships between stakeholders, while proportional piling enabled certain elements to be differentiated by degree of importance [29].

### 2.2 Data synthesis and analysis

All the interviews were transcribed and then analyzed using NVivo 14 software [31] to identify the key ideas and put each statement into context. This software facilitated the systematic organization, coding, and analysis of the data gathered [32]. Quotes from participants were used to illustrate the results and provide insights on the interviewees' viewpoints [33].

### 2.3 Ethics approvals

The study received approval from the National Ethics Committee for Health Research in Guinea as part of the EBO-SURSY project under authorization No. 028/CNERS/22 of April 29, 2022, and No. 050/CNERS/23 of April 5, 2023. At local level, approval was obtained from the local authorities. Before the start of the interviews, a written consent form was signed by each participant or a designated representative for the FGD, specifying that data would be anonymized and only used for research purposes.

## 3 Results

A total of 25 interviews were carried out, including 15 SSIIs and 10 FGDs, for a total of 87 participants, including 26 women and 61 men (Table 1). The average number of participants per FGD was 8.7, ranging from 2 (minimum) to 13 (maximum) people. Flow diagrams were used in 4 FGDs and simple ranking and proportional piling in 2 FGDs. Stakeholders from human health, animal health, environmental health, and civil society were interviewed. These actors belonged to the community level, the subprefectural or prefectural level (or decentralized level), and national level.

**Table 1:** Description of semi-structured interviews (semi-structured individual interviews – SSIIs and focus group discussions – FGDs) conducted in 2022 and 2023 in Forest Guinea. The group in Moussayah was a test focus group.

Date	Location	Type	Health sector	Level	Participants
04/22/2022	Guéckédou	SSII	Animal	Decentralized level	1
04/22/2022	Guéckédou	SSII	Animal	Decentralized level	1
04/23/2022	Guéckédou	SSII	Human	Decentralized level	1
04/24/2022	Temessadou	SSII	Animal	Decentralized level	1
04/24/2022	Temessadou	SSII	Human	Decentralized level	1
04/26/2022	Temessadou	SSII	Environmental	Decentralized level	1
04/27/2022	Guendembou	SSII	Human	Decentralized level	1
04/27/2022	Guendembou	SSII	Environmental	Decentralized level	1
04/28/2022	Guendembou	SSII	Animal	Decentralized level	1
05/04/2022	Conakry	SSII	Human	National level	1
05/05/2022	Conakry	SSII	Environmental	National level	1
03/23/2023	Moussayah	FGD	Mixed	Community	8
04/07/2023	Conakry	FGD	Animal	National level	2
04/14/2023	Temessadou	FGD	Mixed	Community	12
04/152023	Temessadou	FGD	Mixed	Community	10
04/15/2023	Temessadou	FGD	Civil society	Community	2
04/16/2023	Temessadou	FGD	Human	Community	9
04/16/2023	Temessadou	SSII	Animal	Mixed	1
04/17/2023	Temessadou	SSII	Civil society	Community	1
04/18/2023	Guendembou	FGD	Human	Community	8
04/19/2023	Guendembou	FGD	Animal	Community	6
04/19/2023	Guendembou	FGD	Environmental	Community	9
04/20/2023	Guendembou	FGD	Human	Community	6
04/20/2023	Guendembou	SSII	Not related	Community	1
04/20/2023	Guendembou	SSII	Not related	Community	1

Based on the transcript analysis, three topics were identified as most relevant to community-based surveillance: (i) how community healthcare works, (ii) needs for training and awareness, and (iii) financial and logistical barriers.

With regard to the first topic, the interviews provided a detailed overview of the community healthcare network. Each health sector – *i.e.* human health, animal health, and environmental health – has its own community health workers (CHWs) who play a central role in communicating with the local population. The different CHWs are called community human health workers (CHHWs), community animal health workers (CAHWs), and community informants (CIs), who are involved in environmental health. CHHWs help the health center obtain local information on remote populations and to connect with traditional healers. CAWHs, who are trained to identify specific symptoms, are responsible for investigating alerts reported by farmers or concerning domestic animals. CIs are responsible for reporting illegal forest fires or suspicious wildlife deaths, but their activities seem to be less formalized compared to those of the other CHWs. Other stakeholders, such as traditional healers, matrons, and the village announcer, also contribute to community health.

**Fig. 2:**
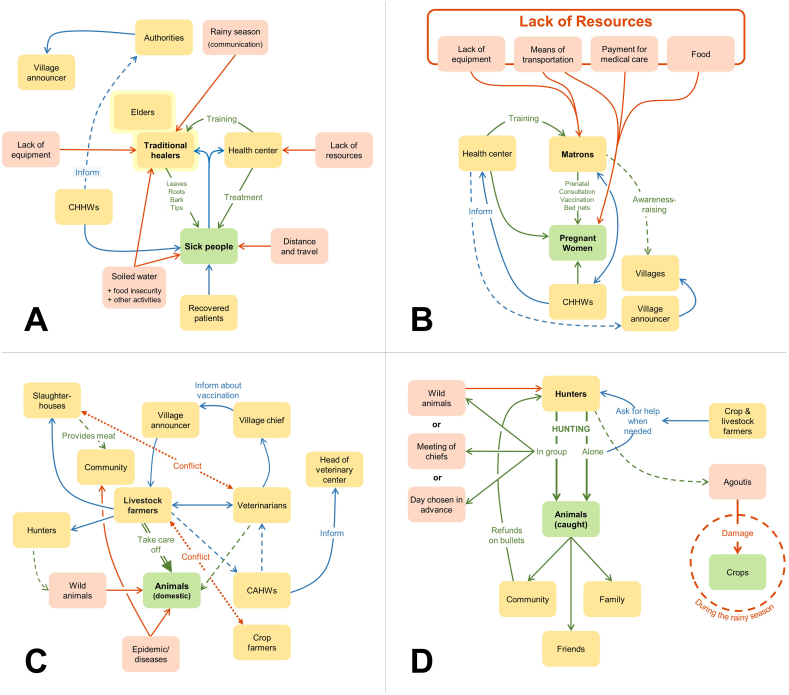
A: Conducted April 20, 2023, with six traditional healers; B: conducted April 18, 2023, with eight matrons in Guendembou, Guéckédou prefecture (CHHWs = community human health workers); C: conducted April 19, 2023, with six livestock farmers in Guendembou; D: conducted April 19, 2023, with nine hunters. Arrows: blue: flow of information; green: beneficial relationship; red: detrimental relationship. Boxes: yellow: stakeholders; green: primary target; light red: causes of problems. Adapted from graphic outputs.

With regard to human health, our interviews showed complementary aspects between conventional and traditional healthcare practices. Traditional healers play a key role in the community healthcare system because of their affordable treatments, their proximity, and the trust that community members place in them. The health center appears to serve more as a reference point for providing training or dealing with disease cases referred by traditional healers (Fig. 2A).

*We have a lot of work to do. In the community, as soon as someone falls, the first thing they say is “We need the healers.” It*'*s us they seek out first. (Traditional healer, translation from kissi)*.

Matrons work with the health center and handle the care of pregnant women (Fig. 2B). They perform both prenatal consultations and deliveries.

*When she [a pregnant woman] goes to the health center, there are specialists there who can observe her, and when it*'*s time for her to give birth, the specialists can call in a matron: “Come, you can help us so that we can do things properly. (Matron, translation from kissi)*.

Livestock farmers are in contact with a large number of community members, and they maintain particularly close relationships with veterinarians and slaughterhouse workers (Fig. 2C). Although they raise different animals (*e.g.*, pigs, cattle, goats), livestock farmers share common activities and concerns. These farmers inform each other when an animal gets sick, and can identify the individual animals based on markings on the skin. Livestock farmers are kept informed of general vaccination campaigns by the village announcer, CAHWs, or the local veterinarian. During the interviews, they mentioned animal diseases as a major concern, but did not associate them with possible contamination of humans or wildlife.

Hunters only consult the health services in the event of serious accident or illness, and did not mention them in the flow diagram (Fig. 2D). They made no mention of CIs. Furthermore, they do not consider zoonotic diseases to be a major risk, whereas they do see bushfires and droughts as alarming events because they can affect the presence of animals (Table 2).

**Table 2:** Simple ranking of risks perceived by the group of hunters consulted in Guendembou, Guinea. Created on 04/19/23.

Ranking	Problems encountered
# 1	Mosquitoes, snakes, flies
# 2	Debt (depending on seasonality)
# 3	Wild animals
# 4	Wildfires
# 5	Accidental shooting
# 6	Unexpected events (getting lost in the woods)
# 7	Sick animals (mentioned first by the interviewer)

Village announcers relay important information to communities, working to reach as many people as possible (Fig. 2). They provide information about health issues, but can also cover other topics. Given that this is generally an unpaid role, their motivation is more personal – they perform this work for the sake of the community.

*When it comes to health, especially vaccination, if I inform the community, a lot of people, I feel good. And that*'*s what motivates me the most. (Village announcer, translation from kissi)*.

Teachers, while not directly involved in healthcare, are also key stakeholders because they give lessons on hygiene and sometimes host awareness-raising campaigns.

Hunters would like to improve the ways they share knowledge between them and benefit from the advice of their elders. Doing so could help them to treat themselves in case of a disease or an injury while hunting in the forest.

*To avoid all these problems [which plants to use, how to avoid mosquitoes, etc…. they want to organize training. There are many of them who know things, but how can they live far away, come, and give training courses? (Hunter, translation from kissi)*.

The lack of knowledge and training among CHWs and CIs is an issue with regard to their activities, because these workers establish a direct link between their own expertise and their ability to raise awareness in the community.

*Once you*'*ve been to one or two of these events [trainings, etc.], it*'*s very easy to give advice and information about it [diseases, etc.]. When we do it, and about personal hygiene too, once you*'*ve already got information about this, you can share this information widely with the community. (Community informant)*.

Participants mentioned several measures to improve communication between those involved in surveillance, early case detection, and community awareness-raising.

*The greatest promoters of disease awareness are the people who have fallen ill and been cured. So if these people find this person or another, they*'*re going to get him or her to join those people [healers, etc.]. (Traditional Healer, translation from kissi)*.

**Fig. 3:**
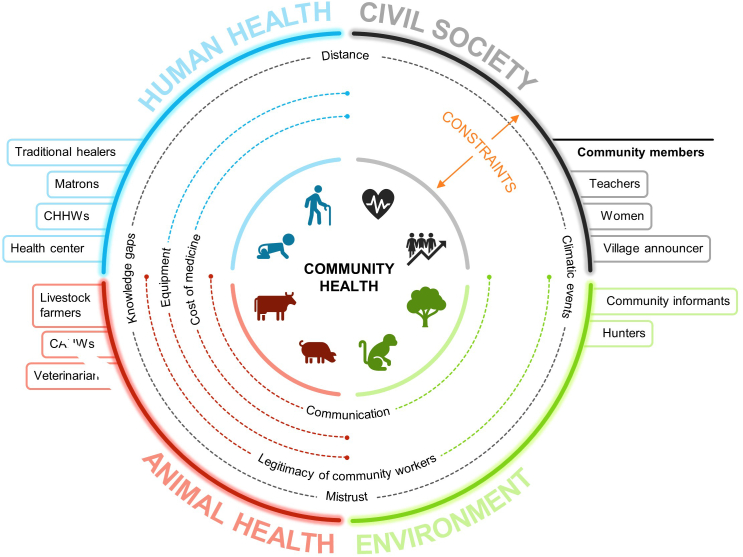
Descriptive diagram of constraints encountered by healthcare sectors and associated stakeholders, as well as community elements of interest.

While participants talked about the importance of awareness and giving health advice to their communities, their efforts are hindered by mistrust or their lack of legitimacy as unofficial authorities. The final topic concerns the financial and logistical barriers faced by community members in carrying out their activities (Fig. 3). During discussions about the flow diagram, the general lack of equipment was a recurring theme.

*Here*'*s the big problem. You*'*re educating a pregnant woman. If she comes to the hospital to give birth, the place where she gives birth is not a convenient place* … *and the equipment is inadequate. (Matron, translation from kissi)*.

Without boots or raincoats, village announcers are particularly affected by rain and humidity during the rainy season. When added to the lack of options to travel between people's workplaces, homes, and the health center, information sharing may be slowed down or may not happen at all. The issue of transportation affects everyone in the community and reduces the number of consultations at the health center, as some members of the community are unable to travel to the center. The price of medications can also be a barrier for community members: they often turn to traditional healers who do not ask for immediate payment. When it comes to livestock farmers, not only are medicines unaffordable, but farmers are also reluctant to have their entire herd vaccinated in order to avoid paying taxes linked to herd size.

The gap in available resources allocated to the different sectors – with human health receiving a larger share than the animal or environmental health sectors – also affects the motivation of CAHWs and CIs, as well as their ability to conduct surveillance activities, although we were not able to gather exact numbers on the issue.

*There are lots of problems, for example in human health they are often paid to go and do training courses, but we get nothing. They promised us telephones, even basic telephones, but we still don*'*t have them. (Head of the veterinary center)*.

## 4 Discussion

This study offers a comprehensive mapping of community health stakeholders and examines the everyday challenges they face, with a focus on healthcare access, information reporting, and disease prevention. Participatory approaches were well-suited to the context: they give a voice to local stakeholders (including citizens), who are the first to be affected by public health decisions but are rarely consulted to develop their own health solutions [17,29].

It is important to acknowledge the apparent difficulty of involving community stakeholders in disease surveillance. Their daily livelihood activities often take precedence, leaving little room for surveillance. There are also other barriers, such as a lack of knowledge about zoonotic diseases, the economic and cultural difficulties related to changing practices that are considered risky [22], and the low importance attached to zoonotic diseases in everyday health, as also observed by Guenin et al. and Drame et al. in Guinea [19,22]. Despite being highly exposed to zoonotic risks, not every community member feels highly concerned by zoonotic diseases. One strategy could be to encourage them to improve their community's health by helping to reduce risky practices or by raising their awareness of zoonotic diseases. In a future study, it would be ideal to interview them directly to hear what role they would like to play in surveillance. Doing so would mean that One Health surveillance must take into account all the local realities of communities exposed to zoonotic risks, since improving general health could also reduce zoonotic risks.

Given the presence of established and active CHWs in the community, the primary focus should be on strengthening systems that are already in place. The ability of CHWs to detect outbreaks seems to depend on a variety of factors, such as motivation, proximity to the communities, or supervision and training [15]. As observed in Yemen, lack of trust between CHWs and investigated communities can affect case reporting rates [34]. Skills to be able to detect an emergence must also be a core part of their training, as it was the case in the 2021 Ebola outbreak in Guinea [7,15,35]. However, the integration between CHWs and subprefectoral level must be operational, with both bottom-up and top-down information flows [36]. Another important aspect is the cross-sectoral approach. As shown in Ghana, the different sectors can improve together by working with a One Health approach [37], which is the case in Guinea at the national level, but the country would benefit from more support at local level [8]. Motivating CHWs is just as important, which is why they should be provided with the equipment needed for surveillance, including awareness-raising tools, telephones, hygiene kits, and equipment specific to the season [15].

This study does have some limitations. The selected participants may not represent the full diversity of their socioprofessional category [38]. However, previous years of on-site research and snowball sampling helped to lower this risk. We also adopted different languages during the FGDs, which could lead to a translation/interpretation bias. Finally, regional differences could mean these results are not equally applicable to the rest of Guinea, even if community stakeholders are similar. Future research carried out on study sites should pay close attention to data that could provide a different perspective.

## 5 Conclusion

This study provides important data for the operationalization of a surveillance system involving community stakeholders. It highlights the determinants of surveillance within a community (beliefs, activities, relations with health services) as well as the difficulties in setting up such a system: lack of resources, disinterest among community stakeholders, lack of trust in community health workers, and the burden of day-to-day activities. These results show the importance of context in setting up a surveillance system, and of establishing it based on the priorities of the primary users. Finally, the strength of relationships and community health also lies in areas where they overlap. This work shows the importance of One Health in community-based surveillance and the need to break the silos between sectors in order to involve as many players as possible in improving outbreak detection.

## CRediT authorship contribution statement

**Maxime Tesch:** Writing – review & editing, Writing – original draft, Visualization, Validation, Software, Resources, Methodology, Investigation, Formal analysis, Data curation, Conceptualization. **Abdoulaye Touré:** Writing – review & editing, Validation, Supervision, Project administration. **Saa André Tolno:** Validation, Methodology, Investigation, Conceptualization. **Hélène De Nys:** Writing – review & editing, Project administration. **Mathieu Bourgarel:** Writing – review & editing, Project administration. **Mamadou Alimou Barry:** Resources. **Mohamed Idriss Doumbouya:** Resources. **Marisa Peyre:** Writing – review & editing, Validation, Supervision, Project administration, Funding acquisition, Conceptualization. **Marie-Marie Olive:** Writing – review & editing, Writing – original draft, Validation, Supervision, Project administration, Methodology, Conceptualization.

## Declaration of competing interest

The authors declare that they have no known competing financial interests or personal relationships that could have appeared to influence the work reported in this paper.

## Data Availability

Data will be made available on request.

## Glossary

Key informant: A resource person who acts as a link between researchers and participants to build trust and provide insights on the local context.
Matron: A female health worker who takes care of pregnant women and children, and who can perform deliveries.
Zoonotic disease or zoonosis: An infectious disease that has jumped from a non-human animal to humans. Zoonotic pathogens may be bacterial, viral, or parasitic.

## Appendix A Supplementary data

Supplementary material Image 1
