# Supplementary material for: Understanding stakeholder relationships and local context to build a community-based one health surveillance system in Guinea
Source: One Health. 2025 Jun 21;21:101117. doi: 10.1016/j.onehlt.2025.101117 (PMC12271907; doi:10.1016/j.onehlt.2025.101117)
Supplement: Supplementary file 1 — Supplementary material [file mmc1.docx]

# Interview guide

Semi-structured interview **Focus group**

# General Aims :

- To understand the group's perception of disease surveillance, with a focus on the language used, communication methods and the importance of alarming health events.
- Define relations with surveillance focal points (CHWs, technical services, veterinarians, etc.)
- Identify the constraints and needs of communities relating to health communication.
- Identify and discuss proposals and ideas from the community regarding their involvement in surveillance.

**Total time:** 1h15’ – 1h30’

# Matériel :

- Recorder and/or telephone
- Notepad
- Post-it notes
- 100 seeds/tokens
- Flipchart
- Felt pens
- Summary of the interview guide

# Introduction :

Nice to meet you, and thank you for your time.

Let me quickly introduce myself, Maxime Tesch, research student in epidemiology. I'm here today as part of my research to try and understand your relationship with disease surveillance. It's not a question of assessing knowledge, or judging practices here. Above all, what's important is that we discuss the communication of health information.

*Introduce other team members, if present.*

In order to study your answers, is it possible to record the interview and take written notes? Anything we discuss will not be associated with your name and will only be used for research purposes. This document summarises what I've just told you. Can we fill it in together? Is that all right?

*Distribute the consent form at the meeting.*

# Theme 1: Communication in health and surveillance

**Primary objective:** Identify the methods of communication for disease surveillance between members of the community, with technical services or with community stakeholders.

# What do you do when alarming events occur that affect the health of you, your relatives or your animals?

# What aspects of health do you communicate about on a daily basis? Are there any special cases in this communication?

# Who do you contact about illnesses? What information is given to whom?

# Why do you communicate information?

# Do you communicate all information relating to diseases? If not, what are the barriers to communication?

# What external factors can affect health surveillance or communication?

# What is your relationship with the technical services regarding these alarming events? Community stakeholders?

# Are there times of year when you are more cautious about diseases?

# Are there times of year when it becomes more difficult to communicate information?

# Theme 2 : Implication et motivation à la surveillance

**Primary objective:** assessing the potential for community-based monitoring: motivation for participation, proposals and solutions, priority issues.

- What place do you give to communication in relation to other health needs here?
- How do you see (possible/future) involvement in disease communication?
- Why would you want to get involved, or why wouldn't you?
- What do you expect in return for your involvement in surveillance?
- Do you think that some practices need to change so that everyone can communicate?
- Is there any information that you would not like to share? If so, for what reasons?
- How do you see the role of communities in surveillance?
- What solutions can you imagine to improve communication about diseases, health and surveillance in general?
- How have you been involved before, and what effect has this had on your practices and health communication?

Tools to use: Flow chart detailing intra-surveillance communication **or** proportional piling about motivations, constraints and expectations **or** seasonal calendar of constraints to surveillance **or** mapping of elements relating to surveillance: location without network / community players / health centre / high-risk locations / etc.

“Do you have any questions?” / Announcing the restitution & co-construction process (future)
